# 

**Published:** 1928

**Authors:** 


### CONTENTS.

| | Page |
|---|---|
| **Indications for Manipulative Surgery.** By A. G. TIMBRELL FISHER, M.C., M.B., B.CH., F.R.C.S. | 165 |
| **The Colloidal State of Matter.** By F. FRANCIS, D.Sc. | 177 |
| **Redundant Colon: A Group of Cases Exhibiting Symptoms Traceable to this Condition.** By GILBERT B. BUSH, B.A., M.B., CH.B., D.M.R.E. (Cantab.) | 181 |
| **The Lymphocytosis of Infection.** By T. WILSON-SMITH, M.D., M.R.C.P. | 187 |
| **The Value of the Chloride Content of the Cerebro-Spinal Fluid in Diagnosis and Prognosis.** By HERBERT ROGERS, M.B., CH.B. | 197 |
| **Meningococcal Septicæmia, with a Report of a Case.** By C. BRUCE PERRY, M.B., M.R.C.P. | 207 |
| **Chemotherapeutic Researches on Cancer—A Review** | 217 |
| **Reviews of Books** | 219 |
| **Editorial Note** | 225 |
| **Meetings of Societies** | 228 |
| **The Medical Library of the University of Bristol** | 230 |
| **Local Medical Notes** | 231 |

